# Standardized serum 25-hydroxyvitamin D concentrations are inversely associated with cardiometabolic disease in U.S. adults: a cross-sectional analysis of NHANES, 2001–2010

**DOI:** 10.1186/s12937-017-0237-6

**Published:** 2017-02-28

**Authors:** Banaz Al-khalidi, Samantha M. Kimball, Michael A. Rotondi, Chris I. Ardern

**Affiliations:** 1 School of Kinesiology and Heath Science, York University, Toronto, M3J1P3 ON Canada; 2 Pure North S’Energy Foundation, Calgary, AB Canada

**Keywords:** Ethnicity, Framingham CVD risk, Insulin resistance, Metabolic syndrome population survey, Standardized 25-Hydroxyvitamin D, Vitamin D

## Abstract

**Background:**

Previously reported associations between vitamin D status, as measured by serum 25-hydroxyvitamin D [25(OH)D] concentrations, and cardiometabolic risk factors were largely limited by variability in 25(OH)D assay performance. In accordance with the Vitamin D Standardization Program, serum 25(OH)D measurement was recently standardized in the National Health and Nutrition Examination Survey (NHANES) to reduce laboratory and method related differences in serum 25(OH)D results. We evaluated the overall and ethnic-specific associations between the newly standardized serum 25(OH)D concentrations and cardiometabolic risk in U.S. adults.

**Methods:**

This study examined standardized 25(OH)D data from five cycles of the NHANES (2001–2010). The total sample included 7674 participants (1794 Mexican-Americans, 4289 non-Hispanic whites, and 1591 non-Hispanic blacks) aged ≥ 20 years who were examined in the morning after overnight fasting. Serum 25(OH)D was directly measured by liquid chromatography-tandem mass spectrometry (LC-MS/MS) in 2007–2010, and was predicted from LC-MS/MS equivalents for 2001–2006. Serum 25(OH)D levels were categorized into quartiles (<43.4, 43.4–58.6, 58.7–74.2, ≥74.3 nmol/L). Cardiometabolic risk was defined by the homeostatic model assessment of insulin resistance (HOMA-IR), metabolic syndrome (MetS), and Framingham cardiovascular disease (CVD) risk. Prevalence ratios and 95% confidence intervals were calculated using modified Poisson regression.

**Results:**

After full adjustment for confounders, serum 25(OH)D ≥74.3 nmol/L was associated with lower cardiometabolic risk compared to 25(OH)D <43.4 nmol/L in the overall sample [HOMA-IR: 0.70 (0.59, 0.84); MetS: 0.82 (0.74, 0.91); CVD risk: 0.78 (0.66, 0.91)]. These associations remained significant in Mexican-Americans [HOMA-IR: 0.54 (0.35, 0.82); MetS: 0.73 (0.55, 0.96)], non-Hispanic whites [HOMA-IR: 0.81 (0.68, 0.96); MetS: 0.84 (0.73, 0.95); CVD risk: 0.78 (0.64, 0.93)]; and in non-Hispanic blacks [HOMA-IR: 0.67 (0.45, 0.99); MetS: 0.75 (0.56, 0.97); CVD risk: 0.58 (0.41, 0.81)].

**Conclusions:**

Low vitamin D status is a significant risk factor for cardiometabolic disease in U.S. adults based on standardized serum 25(OH)D results, irrespective of ethnic background. Future studies using standardized 25(OH)D data are needed to confirm these results, particularly amongst U.S. blacks with 25(OH)D concentrations above 75 nmol/L.

## Background

A role in the renin-angiotensin aldosterone system (RAAS) and extensive immunomodulatory properties have identified vitamin D as a potential modifiable risk factor in cardiometabolic disorders [1–3]. Measurement of vitamin D status is based on circulating total 25-hydroxyvitamin D [25(OH)D] concentrations, which reflect both food intake and endogenous production of vitamin D. Low serum 25(OH)D levels have been linked to a range of non-skeletal health conditions in adults, including metabolic disorders and cardiovascular diseases [4–8]. In addition, vitamin D is thought to play a protective role against the development of type-2 diabetes by improving the insulin secretion of pancreatic beta cells and by maintaining glucose homeostasis [9–13]. However, studies investigating the relation between vitamin D status and cardiometabolic disorders are inconsistent [14–17]. Among the possible explanations for this discrepancy include the substantial heterogeneity among definitions for vitamin D deficiency, different age and ethnic distributions, and large variations in the performance of serum 25(OH)D assays.

Previous analyses of the NHANES have relied on unstandardized serum 25(OH)D measured by the Diasorin radioimmunoassay (RIA) kit, a method that has been criticized for its lack of precision and documented bias [18, 19]. In accordance with the vitamin D Standardization Program (VDSP), the National Center for Health Statistics (NCHS) of the Centers for Disease Control and Prevention (CDC) recently released the standardized serum 25(OH)D data files in October, 2015 [20]. The standardized 25(OH)D data provide the most reliable estimates of serum 25(OH)D concentrations using the ultra-high performance liquid chromatography-tandem mass spectrometry (LC-MS/MS) method [21]. The LC-MS/MS method has improved sensitivity and specificity for serum 25(OH)D metabolites compared to previous immunoassay methods, and the standardization of serum 25(OH)D data allows for comparison across different survey cycles of the NHANES, providing sufficient power to study risk associated with varying concentrations of serum 25(OH)D. Thus, previously reported associations between serum 25(OH)D with cardiometabolic disorders using unstandardized serum 25(OH)D data from previous cycles of NHANES (1988–1994, and 2001–2006) were likely affected by method-related variations in serum 25(OH)D assays.

In addition to the assay-related differences in serum 25(OH)D results, ethnic variations in the relationship between vitamin D status and cardiometabolic disorders have been documented, with mixed results. For example, ethnic-specific differences in diabetes risk by serum 25(OH)D status have been confirmed in previous NHANES cycles (1988–1994, and 2001–2006), where an inverse relationship between unstandardized 25(OH)D concentrations and type-2 diabetes risk was observed in Mexican-Americans and non-Hispanic whites, but not in non-Hispanic blacks [22, 23]. Similarly, 25(OH)D concentrations were significantly associated with fatal stroke and heart failure in NHANES III, with increased risk seen in white participants with low 25(OH)D, but not in black participants [24]. Finally, in a prospective study, an increased risk of coronary heart disease events was reported in white or Chinese participants with low serum 25(OH)D, but not in blacks or Hispanics [25].

Previous analyses using unstandardized data were likely confounded by large variations in serum 25(OH)D results, and ethnic-specific analyses were further constrained by small sample sizes in underrepresented populations, such as U.S. blacks, adding more uncertainty to these estimates. Accurate assessment of the overall and ethnic-specific variations in vitamin D is therefore crucial, and dependent upon the use of standardized data with sufficient statistical power to examine ethnic differences in the relationship between vitamin D status and cardiometabolic risk. Thus, the purpose of this study was to provide a comprehensive assessment of cardiometabolic risk including insulin resistance, metabolic syndrome, and cardiovascular disease risk in relation to serum 25(OH)D levels in U.S. adults, and to estimate the ethnic-specific associations using the newly standardized serum 25(OH)D data from NHANES 2001–2010.

## Subjects and methods

### Participants

Conducted by the NCHS, NHANES is a series of stratified, multistage probability surveys designed to collect cross-sectional data on the health and nutritional status of the civilian, non-institutionalized U.S. population. NHANES is an ongoing survey and data are reported in 2-year intervals, which are available for public use [26]. NHANES oversamples certain under-represented groups in the population, including Mexican Americans, blacks, older adults and those of lower socioeconomic status. Each survey cycle consists of an in-home interview, physical examinations and laboratory tests. Descriptions of the standardized protocols used for data handling during the interview, laboratory, and physical examinations have been previously published [27].

**Fig. 1:**
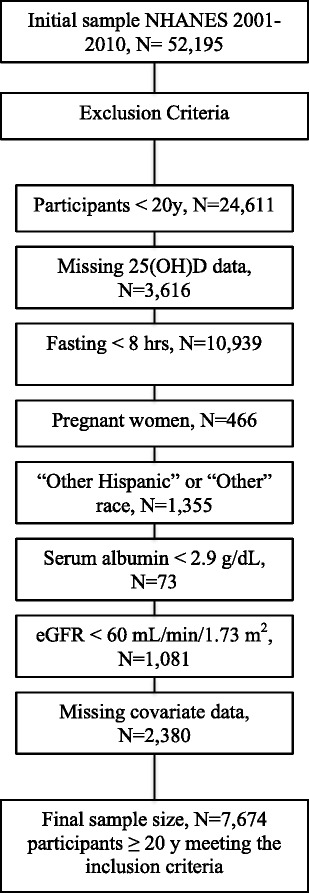
Flowchart showing the exclusion criteria for sample derivation, NHANES 2001–2010

We initially identified 23,968 adults ≥20 years with available standardized serum 25(OH)D data from 2001–2010. We excluded 10,939 participants who fasted < 8 h, 466 pregnant women, 1355 participants in “other Hispanic” or “other race” category, 73 with serum albumin < 2.9 g/dL, 1081 with estimated glomerular filtration rate (eGFR) < 60 mL/min/1.73 m^2^, and 2380 participants with missing covariate information. Those in the “other Hispanic” category were excluded due to a change in the 2007–2010 NHANES sampling design, where Hispanics were oversampled instead of only the Mexican-American population [28]. As such, we only included those in the “Mexican-American” category. The final analytical sample included 7674 adults ≥20 years who fasted for ≥ 8 h and self-identified themselves as Mexican-American (MA), non-Hispanic white (NH-white), or non-Hispanic black (NH-black). A flowchart for study sample derivation is shown in Fig. 1. NHANES was approved by the NCHS institutional board and all adults provided written informed consent [29].

### Measurement of serum 25(OH)D

For 2007–2010, serum 25(OH)D metabolites were analyzed by the CDC laboratory using the LC-MS/MS method and total serum 25(OH)D (nmol/L) was calculated as the sum of 25(OH)D_3_ and 25(OH)D_2_, excluding the C3-epi-25(OH)D_3_ metabolite. For 2001–2006, serum 25(OH)D levels were initially measured using the Diasorin RIA kit (Stillwater, MN); however, due to concerns about bias and imprecision for the Diasorin RIA assay, the CDC developed regression equations to convert RIA values to LC-MS/MS equivalents for NHANES 1988–1994 and 2001–2006 [30]. In order to combine serum 25(OH)D measurements, we used the predicted LC-MS/MS equivalent total serum 25(OH)D data from 2001–2006 and the total serum 25(OH)D data from 2007–2010.

### Outcome variables

#### Insulin resistance

Insulin resistance was estimated by the homeostatic model assessment-insulin resistance index (HOMA-IR), calculated as the product of the fasting insulin concentration (μU/ml) and the fasting plasma glucose concentration (mmol/L), divided by 22.5 [31]. We defined insulin resistance as HOMA-IR ≥ 75^th^ percentile (sex- and ethnic-specific). Fasting glucose concentrations were measured by a Hexokinase enzymatic method, and serum insulin concentrations were measured by a radioimmunoassay in 2001–2002, a two-site immunoenzymometric method in 2003–2004 and an ELISA two-site enzyme immunoassay method in 2005–2010.

#### Metabolic syndrome

We used the revised U.S. National Cholesterol Education Program Adult Treatment Panel III (NCEP/ATPIII) report to define MetS [32] as 3 or more of the following 5 criteria: 1) waist circumference (WC) ≥ 102 cm in men or ≥ 88 cm in women; 2) triglycerides ≥ 1.7 mmol/L or medication; 3) high density lipid (HDL) cholesterol < 1.0 mmol/L in men or < 1.3 mmol/L in women or medication; 4) blood pressure (BP) ≥ 130/85 mmHg or treatment for hypertension; 5) fasting blood glucose ≥ 5.6 mmol/L or treatment for diabetes. WC and BP data were collected during physical examinations and descriptions of the standardized protocol are provided in the NHANES Anthropometry Procedures Manual [33]. Up to four BP readings were obtained during the physical examination and the average BP was estimated using the BP protocol provided by NHANES [34]. Triglycerides were measured enzymatically in serum samples from the morning session. HDL-cholesterol was measured using either the Heparin manganese precipitation method or a direct HDL-cholesterol immunoassay method. Prescription medication use was self-reported during the in-home interview. Using the standardized generic prescription drug codes, medication use was classified into four drug categories; HDL-specific medications, lipid medications, anti-hypertensive medications, and anti-hyperglycemic medications.

#### Framingham CVD Risk

The Framingham CVD risk score was used to estimate the 10-year composite risk for coronary heart disease, stroke, peripheral artery disease, and heart failure [35]. In the first step of the algorithm, point scores for age, sex, total and HDL cholesterol, SBP, treatment of hypertension, smoking, and diabetes status were assigned [35]. CVD risk scores were subsequently computed for each participant based on age- and sex-specific criteria (range for men: −3 to ≥ 18; women: −3 to ≥ 21), which were then translated into a participant’s absolute 10-year risk for a CVD event. The specific details on the point scores for the CVD risk algorithm is provided in Tables five-eight in reference [35]. In our study, an absolute risk of ≥ 15% was defined as “high” and an absolute risk of < 15% as “low” for 10-year predicted risk. These cutoffs were calibrated to approximate the absolute CVD risk associated with insulin resistance (12.6%) and MetS (14.9%) in our study sample.

Total cholesterol was measured enzymatically at Johns Hopkins lipid laboratory (Baltimore, MD). Treatment of hypertension was established from self-reported anti-hypertensive medications. Smoking status (0 = nonsmokers, 1 = current smokers) was self-reported during the in-home interview. Diabetes status was defined as a fasting glucose ≥ 126 mg/dL (7.0 mmol/L) or current use of diabetes medication.

### Confounders

Age, sex, ethnicity, smoking status, educational attainment, physical activity (PA), dietary supplement intake and use of medications were self-reported by questionnaire during the in-home interview. Educational attainment was categorized as less than high school, high school graduate, and some college or college graduate or higher. The PA questionnaire included a series of questions related to participant’s daily activities, leisure-time activities, and sedentary activities at home. Participants self-reported the number of days in the past month or in a typical week they engaged in daily and leisure time activities and the average duration for these activities. A metabolic equivalent (MET) of 4.0 for moderate, and 8.0 for vigorous intensity PA were used to estimate the MET minutes/week for each participant. Supplemental intake of vitamin D for 2007–2010 was obtained from 24-h dietary recall, and total daily vitamin D intake was estimated across all supplement sources for 2001–2006. Vitamin D supplementation was categorized as “any” versus “nonusers”. Season corresponds to season of blood draw, which was reported by NHANES as winter months (November–April) and summer months (May–October). Standing height and weight were measured during the physical examination and were used to calculate body mass index (BMI: kg/m^2^).

### Statistical analysis

For descriptive statistics, weighted means for continuous variables and weighted percentages for categorical variables along with 95% confidence intervals (CIs) were used. Differences in descriptive statistics across ethnic groups were assessed by survey-weighted Wald-F-Test and Chi-square tests to examine the independence of means and frequencies, respectively.

Modified Poisson regression models with robust error variances were used to estimate the prevalence ratios (PRs) and 95% CIs for cohort studies [36, 37]. Serum 25(OH)D concentrations were categorized into quartiles (<43.4, 43.4–58.6, 58.7–74.2, ≥74.3 nmol/L). To calculate the PRs for each outcome, the lowest 25(OH)D quartile (<43.4 nmol/L) was considered as the referent group (PR = 1.00). The models were adjusted for confounders such as age, sex, ethnicity, education, season of blood draw (winter, summer), survey cycle (corresponding to survey year), BMI, vitamin D supplement use, smoking, total PA and relevant medication use (lipid-lowering and anti-hyperglycemic medications). The adjustment of these confounders in the models were based on the association of the confounder variables with both the outcome measures and serum 25(OH)D levels. Pairwise interactions with ethnicity and age were explored by including the product terms in the models (i.e. 25(OH)D*ethnicity and 25(OH)D*age). Models for insulin resistance, MetS, and CVD risk were subsequently stratified by ethnicity, and the CVD risk model was further stratified by age groups (i.e. 20–59 and ≥ 60 years). To explore the possibility of differential bias by comorbid conditions, a series of sensitivity analyses were conducted after the exclusion of participants with physician diagnosed diabetes and CVD (congestive heart failure, coronary hearts disease, angina, heart attack, and stroke), and relevant medication use (i.e. lipid, blood pressure, and anti-hyperglycemic medications).

To ensure the representativeness of the data, clinical fasting weights were applied to account for survey cluster design, oversampling, and nonresponse. Statistical analysis was performed using SAS 9.4 (Cary, NC) survey procedures, which appropriately accounted for cluster sampling and the complex sample design of NHANES. All statistical tests were two-tailed at the alpha = 0.05 level of significance.

## Results

### Participant characteristics

Baseline characteristics of the participants are shown in Table 1. Overall, the mean age of the sample was 45.3 (44.7, 45.9), and the majority of the sample was NH-white (55.9% NH-white, 23.4% MA, and 20.7% NH-black). Of the 7674 participants, 22.5% (21.0, 24.1) were taking vitamin D supplements, with supplement use significantly higher in NH-whites [24.8% (22.8, 26.7)] compared to MAs [13.0% (11.0, 15.0)] and NH-blacks [14.7% (12.1, 17.3)]. The prevalence of MetS in the total sample was 36.1% (34.6, 37.6), and NH-whites had the highest prevalence of MetS [36.9% (35.0, 38.8)] compared to MAs [34.8% (31.5, 38.1)] and NH-blacks [31.6% (29.0, 34.0)]. The absolute CVD risk in the total sample was 7.92% (7.63, 8.20), and NH-Whites had significantly higher absolute CVD risk [8.26% (7.93, 8.59)] compared to MAs [5.85% (5.40, 6.31)] and NH-blacks [7.19% (6.77, 7.61)].

**Table 1:** Population Characteristics of U.S. Adults (≥20 years) in NHANES 2001–2010

	All (*N* = 7674)		Mexican Americans (*N* = 1794)		Non-Hispanic Whites (*N* = 4289)		Non-Hispanic Blacks (*N* = 1591)		*P-value
Age (years)	45.3	44.7, 45.9	38.7	37.8, 39.6	46.6	45.9, 47.3	41.7	40.9, 42.6	<0.0001
BMI (kg/m^2^)	28.5	28.3, 28.6	28.8	28.5, 29.2	28.2	27.9, 28.4	30.1	29.7, 30.5	<0.0001
WC (cm)	97.7	97.3, 98.2	97.1	96.2, 98.0	97.8	97.2, 98.3	98.3	97.3, 99.3	0.225
25(OH)D (nmol/L)	65.5	64.2, 66.9	53.9	52.4, 55.4	70.2	68.9, 71.5	42.2	40.3, 44.0	<0.0001
Winter ^a^	59.4	57.6, 61.3	52.3	50.6, 54.0	65.8	63.9, 67.8	39.8	38.2, 41.3	<0.0001
Summer ^b^	69.4	67.9, 70.9	58.4	55.8, 60.9	72.3	70.8, 73.8	44.9	41.8, 48.1	<0.0001
Supplement nonusers	62.5	61.0, 64.0	52.9	51.3, 54.4	67.4	65.9, 69.0	39.9	38.1, 41.7	<0.0001
Supplement users	75.9	74.2, 77.5	60.5	58.2, 62.7	78.5	76.9, 80.2	55.4	50.8, 59.9	<0.0001
Vitamin D Supplement Use (%)	22.5	21.0, 24.1	13	11.0, 15.0	24.8	22.8, 26.7	14.7	12.1, 17.3	<0.0001
HOMAIR	3.06	2.97, 3.16	3.76	3.52, 4.00	2.93	2.81, 3.05	3.4	3.20, 3.59	<0.0001
MetS (%)	36.1	34.6, 37.6	34.8	31.5, 38.1	36.9	35.0, 38.8	31.6	29.0, 34.0	<0.01
Absolute CVD risk (%)	7.92	7.63, 8.20	5.85	5.40, 6.31	8.26	7.93, 8.59	7.19	6.77, 7.61	<0.0001
Self-reported Diabetes (%)	7.4	6.69, 8.10	8.76	7.33, 10.2	6.85	5.97, 7.74	10.1	8.42, 11.8	<0.01
Self reported CVD (%)	6.34	5.61, 7.08	3.66	2.60, 4.71	6.66	5.79, 7.53	6.34	5.10, 7.59	<0.0001
Medication Use (%)									
Blood Pressure	22.2	20.8, 23.7	9.88	8.26, 11.5	23.4	21.7, 25.1	24.2	22.1, 26.4	<0.0001
Lipid	13.4	12.2, 14.5	6.06	4.65, 7.47	15	13.6, 16.3	8.31	6.90, 9.71	<0.0001
Diabetes	5.2	4.58, 5.82	6.12	5.00, 7.24	4.8	4.06, 5.55	7.24	5.85, 8.61	<0.01
Current smokers (%)	23.8	22.3, 25.4	19.1	16.7, 21.4	24.2	22.4, 26.1	24.8	22.2, 27.4	<0.01
Education (% College)	58	55.8, 60.2	28.1	25.0, 31.1	62.6	59.7, 65.4	50.5	47.5, 53.4	<0.0001

Values are weighted means or frequencies (%), and 95% CIs. *P values are based on Wald F-Test or Chi-square test, which test the independence of means and frequencies across ethnic groups

^a^ Participants sampled from November–April. ^b^ Participants sampled from May–October

### Serum 25(OH)D and HOMA-IR

The adjusted PRs for cardiometabolic risk by 25(OH)D quartiles in the total sample are shown in Table 2. After adjusting for age, sex, BMI, PA, ethnicity, season, survey cycle, education, smoking, and use of vitamin D supplement, lipid-lowering and anti-hyperglycemic medications, the highest 25(OH)D quartile (≥74.3 nmol/L) had a 30% relative risk reduction in HOMA-IR [0.70 (0.59, 0.84)] compared to those in the lowest quartile (<43.4 nmol/L). The associations remained significant across all ethnic groups (Table 3). The highest relative risk reduction was observed in MAs [0.54 (0.35, 0.82)], followed by NH-blacks [0.67 (0.45, 0.99), and NH-whites [0.81 (0.68, 0.96)] with serum 25(OH)D ≥74.3 nmol/L compared to the lowest 25(OH)D quartile (<43.4 nmol/L).

**Table 2:** Prevalence ratios of cardiometaolic risk associated with serum 25(OH)D status in U.S. adults, NHANES 2001–2010

	HOMAIR				MetS				CVD Risk (≥15%)			
	Yes	No	PR	95% CI	Yes	No	PR	95% CI	Yes	No	PR	95% CI
25(OH)D quartiles (nmol/L)												
< 43.4	656	1231	Ref		853	1034	Ref		487	1400	Ref	
43.4–58.6	529	1365	1.02	0.89, 1.17	825	1069	1.04	0.95, 1.13	495	1399	0.86 ^*^	0.76, 0.98
58.7–74.2	444	1528	0.89	0.76, 1.04	835	1137	1.00	0.90, 1.10	553	1419	0.90	0.79, 1.02
≥ 74.3	289	1632	0.70 ^*^	0.59, 0.84	630	1291	0.82 ^*^	0.74, 0.91	430	1491	0.78 ^*^	0.66, 0.91
^a^ Excluding those with self-reported diabetes, CVD, and taking medications (lipid, diabetes, blood pressure)												
25(OH)D quartiles (nmol/L)												
< 43.4	347	890	Ref		361	876	Ref		150	1087	Ref	
43.4–58.6	272	993	0.88	0.73, 1.06	350	915	0.99	0.84, 1.15	139	1126	0.85	0.64, 1.14
58.7–74.2	192	1076	0.83	0.66, 1.03	330	938	0.97	0.80, 1.17	141	1127	0.77	0.57, 1.04
≥ 74.3	108	1145	0.60 ^*^	0.45, 0.81	193	1060	0.65 ^*^	0.52, 0.80	119	1134	0.64 ^*^	0.47, 0.87

Models are adjusted for age, sex, education, ethnicity, season of blood draw, survey cycle, smoking, BMI, total physical activity, vitamin D supplement use, and lipid and anti-hyperglycemic medications

^a^ In sensitivity analyses, models are adjusted for age, sex, education, ethnicity, season of blood draw, survey cycle, smoking, BMI, total physical activity, and vitamin D supplement use

^*^ *P* < 0.05

**Table 3:** Ethnic-specific prevalence ratios for cardiometabolic risk according to serum 25(OH)D status, NHANES 2001–2010

	HOMAIR				MetS				CVD Risk (≥15%)			
	Yes	No	PR	95% CI	Yes	No	PR	95% CI	Yes	No	PR	95% CI
A												
*Mexican Americans*												
25(OH)D quartiles (nmol/L)												
< 43.4	175	346	Ref		263	258	Ref		122	399	Ref	
43.4–58.6	156	445	0.9	0.70, 1.15	253	348	0.80 ^*^	0.66, 0.97	132	469	0.85	0.66, 1.09
58.7–74.2	94	366	0.82	0.63, 1.07	188	272	0.95	0.78, 1.16	109	351	0.87	0.67, 1.14
≥ 74.3	25	187	0.54 ^*^	0.35, 0.82	67	145	0.73 ^*^	0.55, 0.96	45	167	0.90	0.69, 1.16
*Non-Hispanic Whites*												
25(OH)D quartiles (nmol/L)												
< 43.4	178	274	Ref		255	197	Ref		156	296	Ref	
43.4–58.6	290	623	1.33	0.94, 1.36	452	461	1.07	0.95, 1.21	271	642	0.86	0.73, 1.01
58.7–74.2	314	998	0.93	0.77, 1.24	565	747	1.00	0.88, 1.14	378	934	0.88	0.75, 1.04
≥ 74.3	290	1322	0.81 ^*^	0.68, 0.96	528	1084	0.84 ^*^	0.73, 0.95	364	1248	0.78 ^*^	0.64, 0.93
*Non-Hispanic Blacks*												
25(OH)D quartiles (nmol/L)												
< 43.4	246	668	Ref		327	587	Ref		209	705	Ref	
43.4–58.6	80	300	0.89	0.71, 1.11	115	265	1.00	0.84, 1.19	92	288	0.83	0.65, 1.05
58.7–74.2	53	147	1.03	0.78, 1.38	78	122	1.11	0.89, 1.37	66	134	0.98	0.78, 1.23
≥ 74.3	18	79	0.67 ^*^	0.45, 0.99	34	63	0.74 ^*^	0.56, 0.97	21	76	0.58 ^*^	0.41, 0.81
B Excluding participants with self-reported diabetes, CVD, and those taking relevant medications												
*Mexican Americans*												
25(OH)D quartiles (nmol/L)												
< 43.4	98	273	Ref	135	236	Ref	41	330	Ref			
43.4–58.6	89	343	0.99	0.72, 1.37	124	308	0.82	0.63, 1.07	32	400	0.49 ^*^	0.29, 0.82
58.7–74.2	56	289	0.98	0.69, 1.38	106	239	1.08	0.82, 1.41	37	308	0.81	0.46, 1.44
≥ 74.3	15	143	0.53 ^*^	0.31, 0.91	31	127	0.63 ^*^	0.44, 0.91	17	141	1.27	0.83, 1.94
*Non-Hispanic Whites*												
25(OH)D quartiles (nmol/L)												
< 43.4	86	166	Ref	98	154	Ref	46	206	Ref			
43.4–58.6	146	436	0.92	0.73, 1.19	184	398	0.98	0.79, 1.22	83	499	0.78	0.54, 1.13
58.7–74.2	124	692	0.76 ^*^	0.58, 0.99	198	618	0.89	0.71, 1.12	92	724	0.68 ^*^	0.47, 0.97
≥ 74.3	105	931	0.61 ^*^	0.44, 0.83	156	880	0.61 ^*^	0.47, 0.80	96	940	0.56 ^*^	0.39, 0.80
*Non-Hispanic Blacks*												
25(OH)D quartiles (nmol/L)												
< 43.4	126	488	Ref	128	486	Ref	63	551	Ref			
43.4–58.6	39	212	0.89	0.64, 1.24	42	209	1.05	0.77, 1.45	24	227	1.05	0.70, 1.58
58.7–74.2	19	88	1.1	0.69, 1.76	26	81	1.49	1.06, 2.10	12	95	1.26	0.66, 2.38
≥ 74.3	5	54	0.73	0.32, 1.70	6	53	0.55	0.25, 1.21	6	53	0.72	0.33, 1.58

Models are adjusted for age, sex, education, season of blood draw, survey cycle, smoking, BMI, total physical activity, vitamin D supplement use, and lipid and anti-hyperglycemic medications. ^*^ *P* < 0.05

### Serum 25(OH)D and MetS

Overall, serum 25(OH)D levels ≥74.3 nmol/L were associated with a 18% risk reduction in MetS [0.82 (0.74, 0.91)] compared to the lowest 25(OH)D quartile (Table 2). The association of serum 25(OH)D with MetS remained significant across all ethnic-groups (Table 3). For MAs, the second 25(OH)D quartile (Q2: 43.4–58.6 nmol/L) had a 20% relative risk reduction in MetS [0.80 (0.66, 0.97) and the highest quartile a 27% risk reduction in MetS [0.73 (0.55, 0.96)] compared to the lowest quartile. Significant associations were observed only in the highest 25(OH)D quartile in NH-whites [0.84 (0.73, 0.95)] and NH-blacks [0.74 (0.56, 0.97)] compared to the lowest quartile.

### Serum 25(OH)D and CVD risk

In the overall sample, the relative reduction in CVD risk was 14% in the second 25(OH)D quartile [0.86 (0.76, 0.98)], and 22% in the highest versus lowest 25(OH)D quartile [0.78 (0.66, 0.91)] (Table 2). In ethnic-specific analyses, significant associations were observed amongst the highest versus lowest quartiles for NH-whites [0.78 (0.64, 0.93)] and NH-blacks [0.58 (0.41, 0.81)], but not in MAs [0.90 (0.69, 1.16)] (Table 3).

**Fig. 2:**
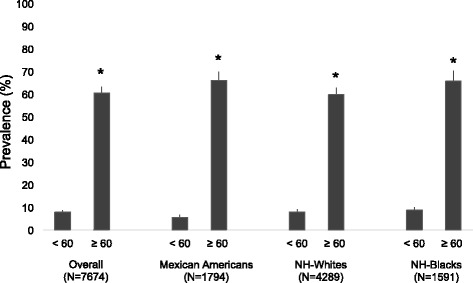
Prevalence of cardiovascular disease risk (≥15%) by age status in participants ≥ 20 years, NHANES 2001–2010. *P < 0.05

There was a significant interaction between serum 25(OH)D and age in the CVD risk model. This is consistent with the Framingham CVD risk because age is the most heavily weighted variable in the algorithm, as shown in Fig. 2. Therefore, we further stratified the association of serum 25(OH)D with CVD risk by age groups (20–59 and ≥ 60 years). Table 4 shows the adjusted PRs of CVD risk associated with 25(OH)D quartiles by age category. Overall, stratification by age resulted in similar results compared to the main model, except that the PRs for CVD risk in participants for 20–59 years old were lower compared to participants ≥ 60 years old. Age stratification did not affect the observed non-significant association of 25(OH) with CVD risk in MAs. For NH-blacks, significant associations were attained in participants ≥ 60 years for the highest quartile [0.63 (0.45, 0.870], but no significant association was found in participants 20–59 years old.

**Table 4:** Adjusted prevalence ratios of cardiovascular risk (≥15%) associated with serum 25(OH)D by age, NHANES 2001–2010

			CVD risk (≥15%)					
Serum 25(OH)D quartiles (nmol/L)		Q1 (<43.4)	Q2 (43.4–58.6)		Q3 (58.7–74.2)		Q4 (≥74.3)	
Overall	20–59 (y)	Ref	0.78	0.60, 1.02	0.93	0.70, 1.23	0.65^*^	0.47, 0.92
	≥60 (y)	Ref	0.99	0.89, 1.09	0.89^*^	0.80, 0.99	0.82^*^	0.73, 0.92
^a^ Mexican Americans	20–59 (y)	Ref	0.70	0.49, 1.01	0.80	0.50, 1.27	0.75	0.44, 1.29
	≥60 (y)	Ref	0.93	0.79, 1.10	0.92	0.78, 1.08	0.81	0.63, 1.03
^a^ Non-Hispanic Whites	20–59 (y)	Ref	0.70	0.48, 1.02	0.89	0.62, 1.29	0.61^*^	0.40, 0.93
	≥60 (y)	Ref	1.01	0.88, 1.16	0.90	0.79, 1.04	0.82^*^	0.74, 0.97
^a^ Non-Hispanic Blacks	20–59 (y)	Ref	1.03	0.75, 1.43	0.97	0.69, 1.37	0.51	0.19, 1.40
	≥60 (y)	Ref	0.88	0.75, 1.04	0.86	0.71, 1.04	0.63^*^	0.45, 0.88

Models are adjusted for age, sex, education, ethnicity, season of blood draw, survey cycle, smoking, BMI, total physical activity

vitamin D supplement use, and lipid and anti-hyperglycemic medications. ^*^ *P* < 0.05

^a^ The ethnicity covariate is excluded in the ethnic-specific models

### Sensitivity analyses

After excluding participants with comorbid conditions, the estimates of PRs for the adjusted associations of 25(OH)D with HOMA-IR, MetS and CVD risk remained statistically significant (Table 2). Similarly in the ethnic-specific analyses, the adjusted associations of 25(OH)D with HOMA-IR, MetS and CVD risk were numerically similar in MAs and NH-Whites after excluding participants with comorbid conditions, although the associations were attenuated for NH-Blacks due to significant reductions in the number of events, particularly in the highest serum 25(OH)D quartile (Table 3). Overall, the sensitivity analyses revealed the stability of the PRs estimates in the original models.

## Discussion

In this study, we have shown that standardized serum 25(OH)D concentrations were inversely associated with insulin resistance, MetS and CVD risk in a nationally representative sample of U.S. adults. These associations remained significant across all ethnic subgroups, except for CVD risk in MAs. Overall, the results of our study suggest that serum 25(OH)D concentrations ≥ 75 nmol/L may be the optimal threshold in relation to cardiometabolic risk. To our knowledge, this study provides the most recently updated and comprehensive estimate of the association between standardized serum 25(OH)D levels and cardiometabolic risk, and unlike previous studies, our findings demonstrate that low 25(OH)D is a significant risk factor for cardiometabolic risk in U.S. blacks.

The inverse associations between vitamin D status and insulin resistance, MetS and CVD risk are aligned with previous epidemiologic studies. Of note, previous analyses of NHANES have found an inverse association between unstandardized serum 25(OH)D levels with insulin resistance and pre-diabetes [11, 38], MetS [4], and CVD mortality [39]. Similarly, prospective studies have shown an inverse association between serum 25(OH)D and insulin resistance [40] and incident CVD in the Framingham Offspring Study [5], and incidence of MetS in a cohort of non-diabetic adults [8].

Previous studies have shown ethnic differences in the association of serum 25(OH)D with cardiometabolic risk factors. Ethnic differences between serum 25(OH)D and risk of diabetes were found in NHANES III [22], and confirmed in NHANES 2001–2006 with significant inverse associations in MAs and NH-whites, but not in NH-Blacks [23]. In a prospective study, Robinson-Cohen et al. [25] reported a significant increase in the risk of coronary heart disease events with low 25(OH)D levels in whites and Chinese, but not in black or Hispanic participants from the Multi-Ethnic Study of Atherosclerosis. Lutsey et al. [41] also observed that low 25(OH)D levels were a stronger risk factor for the development of heart failure in whites than black participants from the Atherosclerosis Risk in Communities study. Conversely, our results showed a significant inverse association of standardized 25(OH)D with cardiometabolic risk in MAs, NH-Whites, and NH-blacks.

Our results are in contrast to the vitamin D paradox observed in blacks, where prior studies have suggested that even though blacks have circulating 25(OH)D in the deficient range (<50 nmol/L), a compensatory mechanism exists in relation to bone health [42, 43]. At this time, it is not fully understood whether other compensatory mechanisms exist in relation to cardiovascular health outcomes, but it has been speculated that vitamin D binding protein (DBP) levels in blacks are significantly lower compared to whites to compensate for lower total serum 25(OH)D in blacks. It has also been suggested that total 25(OH)D may not be the best biomarker of vitamin D status in blacks [44]. However, the monoclonal immunoassay used by Powe et al. [44] has been criticized for its lack of sensitivity to DBP polymorphisms, which provides erroneous results for vitamin D metabolites [45]. Using a novel LC-MS/MS method, Henderson et al. [46] recently showed that levels of DBP do not vary between whites and blacks. Therefore, it is possible that the use of immunoassays in a population with a low range of vitamin D metabolites results in inaccurate total 25(OH)D concentrations in blacks and that the results from existing immunoassays might be insufficient to allow identification of a significant association within these populations.

Previous attempts to assess the ethnic-specific association between cardiometabolic disorders and 25(OH)D have been limited due to reliance on samples with a low number of blacks within the upper range of 25(OH)D concentrations (i.e. ≥ 75 nmol/L). In comparison with prior work, our results suggest significant risk reductions in NH-blacks, which we speculate is due to reduced variation from the standardization of 25(OH)D measurements across all NHANES survey cycles [30]. Moreover, the lowest levels of 25(OH)D were observed in NH-blacks in our study, supporting the hypothesis that black Americans have reduced cutaneous synthesis of vitamin D due to increased skin pigmentation and decreased sun exposure [47]. In support of this hypothesis, Alzaman et al. [48] did not observe any systemic difference in the absorption or bioavailability of oral vitamin D metabolites between US blacks and whites, neither do their results support the paradoxical clinical correlates of vitamin D in US blacks [48]. Taken together, our results add to the current evidence by demonstrating that total circulating 25(OH)D is a significant biomarker of cardiometabolic risk in U.S. blacks.

Although mechanisms relating vitamin D deficiency and cardiometabolic risk factors are not fully elucidated, several important mechanisms warrant discussion. Insulin resistance is the core trait of the metabolic disturbances observed in MetS, type-2 diabetes and CVD risk [49]. The presence of vitamin D receptors (VDRs) in pancreatic β-cells suggest that the active metabolite 1-α-25-dihydroxyvitamin D_3_ (1,25(OH)_2_D_3_) could directly influence insulin secretion [50]. Since insulin secretion is a calcium dependent process, it is possible that 1,25(OH)_2_D_3_ modulates insulin secretion by increasing intracellular calcium levels [50]. Moreover, vitamin D deficiency promotes secondary hyperparathyroidism, stimulates the RAAS, which in turn increases the secretion of aldosterone [51]. Both low serum 25(OH)D and high PTH levels are related to arterial stiffness and vascular dysfunction, which are significant elements of hypertension and CVD risk [52]. It is possible that lower 25(OH)D and higher serum PTH levels could synergistically or independently play a role in the pathogenesis of developing hypertension and future CVD [53]. Due to the lack of data in our study, we were not able to elucidate the role of PTH in our analyses, however, the exclusion of participants with eGFR < 60 mL/min/1.73 m^2^ reduces the potential confounding of secondary hyperparathyroidism caused by chronic renal failure and the impaired vitamin D metabolism by the diseased kidneys. Future studies using standardized serum 25(OH)D data are needed to re-evaluate the optimal 25(OH)D thresholds that maximally suppress PTH levels [54]. The complexity of vitamin D and PTH metabolism makes it challenging to fully disentangle the individual contributions of these factors and warrants further investigation.

In addition, the relationship between vitamin D deficiency and metabolic traits of these diseases is confounded by adiposity where individuals with obesity have lower serum 25(OH)D levels compared to normal-weight individuals and this is consistent across different age and ethnic groups [55–58]. Hence, serum 25(OH)D concentrations are related to fat mass and changes in serum PTH, and adipokines, such as leptin and adiponectin, may be crucial in elucidating the relationship between vitamin D, PTH and metabolic disturbances [59, 60]. It is possible that normal vitamin D metabolism may be disrupted in obesity due to elevated levels of serum leptin, which has been shown to suppress the conversion of 25(OH)D to 1,25(OH)_2_D_3_ [61]. Although we were not able to further adjust for leptin levels, we adjusted for BMI, which is a proxy for fat mass and leptin levels [62]. Moreover, given that obesity and metabolic diseases are associated with low-grade inflammation, the anti-inflammatory and immunomodulatory properties of vitamin D have been suggested and supported by previous studies showing an inverse, but inconsistent, relationship between vitamin D and inflammatory markers such as C-reactive protein (CRP) [63–65]. A recent Mendelian randomization study found no causal relationship between vitamin D and CRP [66], which suggests that it is unlikely that vitamin D deficiency directly contributes to increased inflammation or vice versa. In our study, we found no evidence of the potential confounding effect of CRP levels on the relationship between serum 25(OH)D and all outcome measures (data not shown). Taken together, experimental and observational studies suggest that the relationship between vitamin D and cardiometabolic disorders are multimodal and mediated through direct and indirect pathways. Further, it is important that the results of this study are interpreted according to variation in vitamin D metabolism genes, which could explain the heterogeneity of responses to vitamin D deficiency across ethnic groups [67].

Although the evidence from previous observational studies and our results suggest that serum 25(OH)D levels ≥ 75 nmol/L are associated with lower risk of cardiometabolic disorders compared to 25(OH)D ≤ 50 nmol/L, these findings have not been consistent in clinical trials [68–71]. Among the possible explanations for these differences include the substantial heterogeneity in the definition of vitamin D deficiency, different age structure and target population (i.e., vitamin D deficient versus sufficient individuals), primary versus secondary prevention of CVD, differences in the available assays used to measure vitamin D metabolites, as well as the inclusion of different confounders in their analyses. Although the relationship between optimal vitamin D status and cardiovascular health remains to be elucidated, the standardization of serum 25(OH)D data and the large sample size of our study allowed a comprehensive estimate of the association between 25(OH)D and cardiometabolic risk in US adults using a nationally representative survey sample. Given that many risk factors for CVD are clustered in insulin resistance and MetS, it is reasonable to speculate that low 25(OH)D is a significant risk factor in their development, which may ultimately contribute to increased CVD risk. Future studies must be sufficiently powered to estimate the overall benefit, as well as study the risks and benefits associated with varying circulating 25(OH)D concentrations in ethnic subpopulations.

There are several limitations to this study. Due to the cross-sectional design, we are unable to rule out the possibility of reverse causation. Although we have adjusted for potential confounders, we cannot rule out residual confounding or the effect of unmeasured confounders. Further, seasonality of serum 25(OH)D levels in NHANES must be considered a confounder because of the sampling design wherein data is collected from northern states in the summer and southern states in the winter. This sampling design results in higher average 25(OH)D and an increased range of 25(OH)D concentrations in northern states. Despite these limitations, our study provides national estimates of the association between serum 25(OH)D and cardiometabolic risk in the U.S. population and in ethnic subgroups using standardized serum 25(OH)D data from 2001–2010.

## Conclusions

In this large national sample, standardized serum 25(OH)D concentrations were significantly associated with cardiometabolic risk including insulin resistance, MetS and CVD risk in U.S. adults, and low 25(OH)D is a significant risk factor for cardiometabolic risk in MAs, NH-whites, and NH-blacks. Standardization of serum 25(OH)D allows accurate assessment of vitamin D status, and future studies are needed to re-evaluate these risks in black Americans with serum 25(OH)D concentrations above 75 nmol/L.

## Abbreviations

1,25(OH)_2_D_3_: 1-α-25-dihydroxyvitamin D_3_
25(OH)D: Serum 25-hydroxyvitamin D
CDC: Center for Disease Control and Prevention
CI: Confidence interval
CRP: C-reactive protein
CVD: Cardiovascular disease
eGFR: Estimated glomerular filtration rate
HOMA-IR: Homeostatic Model Assessment of Insulin Resistance
LC-MS/MS: Liquid Chromatography-tandem Mass Spectrometry
MA: Mexican-American
MetS: Metabolic Syndrome
NCHS: National Center for Health Statistics
NHANES: National Health and Nutrition Examination Survey
NH-black: Non-hispanic black
NH-white: Non-hispanic white
PR: Prevalence ratio
PTH: Parathyroid hormone
RAAS: Renin-angiotensin aldosterone system
RIA: Radioimmunoassay
VDSP: Vitamin D Standardization Program
