# Erratum to: Standardized serum 25-hydroxyvitamin D concentrations are inversely associated with cardiometabolic disease in U.S. adults: a cross-sectional analysis of NHANES, 2001–2010

**DOI:** 10.1186/s12937-017-0251-8

**Published:** 2017-05-22

**Authors:** Banaz Al-khalidi, Samantha M. Kimball, Michael A. Rotondi, Chris I. Ardern

**Affiliations:** 1 School of Kinesiology and Heath Science, York University, Toronto, M3J1P3 ON Canada; 2 Pure North S’Energy Foundation, Calgary, AB Canada

## Erratum

After the publication of this article [1] it was noticed that Table 3B was misaligned.

In Table 3B in both the PDF and online versions: **Mexican Americans**- the numbers "135, 236, Ref, 41, 330, Ref" should be shifted to the right (i.e., **"135"** should read under **MetS "Yes"** column; **"236"** under **MetS "No"** column; **"Ref"** under **"PR"** column for **MetS** ; **"41"** under **CVD risk (>15%) "Yes"** column; **"330"** under **CVD risk (>15%) "No"** column; and the final **"Ref"** under **"PR"** column for **CVD risk**.

**Non-Hispanic Whites**- the numbers "98, 154, Ref, 46, 206, Ref" should be shifted to the right (i.e., **"98"** should read under **MetS "Yes"** column; **"154"** under **MetS "No"** column; **"Ref"** under **"PR"** column for **MetS** ; **"46"** under **CVD risk (>15%) "Yes"** column; **"206"** under **CVD risk (>15%) "No"** column; and the final **"Ref"** under **"PR"** column for **CVD risk**.

**Non-Hispanic Blacks**- the numbers "128, 486, Ref, 63, 551, Ref" should be shifted to the right (i.e., **"128"** should read under **MetS "Yes"** column; **"486"** under **MetS "No"** column; **"Ref"** under **"PR"** column for **MetS** ; **"63"** under **CVD risk (>15%) "Yes"** column; **"551"** under **CVD risk (>15%) "No"** column; and the final **"Ref"** under **"PR"** column for **CVD risk**.

The original article was corrected.

The publisher apologises for these errors.
